# Overexpression of Calretinin Enhances Short-Term Synaptic Depression

**DOI:** 10.3389/fncel.2019.00091

**Published:** 2019-03-13

**Authors:** Alexey P. Bolshakov, Alexander Kolleker, Evgenia P. Volkova, Fliza Valiullina-Rakhmatullina, Peter M. Kolosov, Andrei Rozov

**Affiliations:** ^1^Institute of Higher Nervous Activity and Neurophysiology, Russian Academy of Sciences (RAS), Moscow, Russia; ^2^Research Laboratory of Electrophysiology, Pirogov Russian National Research Medical University, Moscow, Russia; ^3^Max Planck Institute for Medical Research, Department of Molecular Neurobiology, Heidelberg, Germany; ^4^Buryat State University, Medical Institute, Ulan-Ude, Russia; ^5^Laboratory of Neurobiology, Institute of Fundamental Medicine and Biology, Kazan Federal University, Kazan, Russia; ^6^Department of Physiology and Pathophysiology, University of Heidelberg, Heidelberg, Germany

**Keywords:** interneuron, calretenin, release probability, viral expression, pyramidal cells, neocortex

## Abstract

Analysis of the effects of various proteins on short-term synaptic plasticity is a difficult task, which may require the use of knockout animals. Here, we propose an alternative experimental approach for studying the roles of desired proteins in synaptic plasticity. We packed the Ca^2+^-binding protein calretinin and the fluorescent protein Venus into AAV and injected the concentrated viral suspension into the neocortex of newborn rats. The infected layer 2/3 pyramidal cells were identified in rat cortical slices using Venus fluorescence. Analysis of short-term synaptic plasticity using paired patch clamp recordings between layer 2/3 pyramidal cells (presynaptic cell) and fast-spiking (FS) interneurons (post-synaptic cell) showed that calretinin expression in the pyramidal cells did not change the failure rate in this synapse but did decrease synaptic delay. Analysis of the parameters of short-term synaptic plasticity showed that the amplitude of the first EPSP in the train was not affected by calretinin, however, calretinin strongly enhanced short-term depression. In addition, we found that the effect of calretinin depended on the presynaptic firing frequency: an increase in frequency resulted in enhancement of synaptic depression.

## Introduction

A large number of cytosolic proteins have the capacity to bind Ca^2+^, however, traditionally three of them, parvalbumin, calbindin, and calretinin, are considered to act as the main Ca^2+^-endogenous buffers. These proteins differ by Ca^2+^-affinity, expression pattern, and binding kinetics, which determine their effect on synaptic efficacy and plasticity. Analysis of their functional role may be quite challenging because some of these proteins are predominantly expressed in interneurons constituting a relatively small neuronal population (<20%), which is further subdivided into numerous subtypes. Each subtype has its own unique connectivity pattern, synaptic properties, and Ca^2+^-endogenous buffer expression profile. It would not be an exaggeration to say that for the vast majority of interneurons most of these parameters are not well studied when compared to the excitatory synapses formed by cortical glutamatergic neurons. The second complication arises from the classical investigation approach of the function of specific gene products by comparing WT and knockout animals. In the case of endogenous Ca^2+^ buffers, this should be done on connections where the identities of both pre- and post-synaptic neurons are constant in all experiments, this requirement greatly narrows experimental conditions. Moreover, it has been shown that the removal of some Ca^2+^-binding proteins has more global consequences than simply reduction of endogenous buffer capacity. For instance, parvalbumin knockout leads to changes in short-term plasticity from depression to facilitation (Caillard et al., 2000), but also affects mitochondrial morphology (Henzi and Schwaller, 2015; Lichvarova et al., 2018). Ideally, the effects of chronic deletion of endogenous Ca^2+^ buffers should be confirmed by wash-in experiments, where exogenously loaded buffer rescues the affected function (Blatow et al., 2003). In the case of calretinin, these research obstacles are multiplied by the fact that cortical interneurons expressing this protein predominantly innervate other GABAergic interneurons (Caputi et al., 2009). Consequently, both pre- and post-synaptic cells belong to “neuronal minorities,” which make quite difficult to reliably find connected neuron pairs. However, the biophysical rules, by which endogenous buffers shape Ca^2+^ dynamics, depend on Ca^2+^ binding properties rather than the identity of the synapse. Indeed, partial activity-dependent saturation of calbindin underlies facilitation in both neocortical GABAergic and hippocampal glutamatergic terminals (Blatow et al., 2003). Thus, artificially expressed calretinin in terminals with initially low buffer capacity should affect intracellular Ca^2+^ concentration ([Ca^2+^]_i_) in a similar way as it does in WT calretitin-positive interneurons. Pyramidal cells in layer 2/3 of the rat somatosensory cortex have low endogenous Ca^2+^ capacity and some of the excitatory connections formed by these neurons are very well characterized (Rozov et al., 2001). Therefore, in order to study the effect of calretinin on synaptic release, the protein was virally introduced into these cells. As the “calretinin knockout neurons” we used either Venus-negative cells in the same slice or/and layer 2/3 pyramidal cells in slices from non-infected rats. Applying this approach we characterized the effects of calretinin on synaptic efficacy and plasticity at one of the most well studied neocortical synapses formed by presynaptic layer 2/3 pyramidal cells and postsynaptic fast-spiking (FS) interneurons (Gupta et al., 2000; Koester and Sakmann, 2000; Rozov et al., 2001; Blatow et al., 2003; Holmgren et al., 2003; Watanabe et al., 2005; Neske et al., 2015; Pala and Petersen, 2015; Voinova et al., 2015).

## Materials and Methods

All experiments with animals were performed in line with the Russian rules regulating the use of animals for experimental studies and the European Communities Council Directive 86/609/EEC. The protocol of the experiment was approved by the Ethical Commission of the Institute of Higher Nervous Activity and Neurophysiology of the Russian Academy of Sciences.

### Cloning of the pAAV-Syn-Calr-IRES-Venus Plasmid

**Figure 1:**
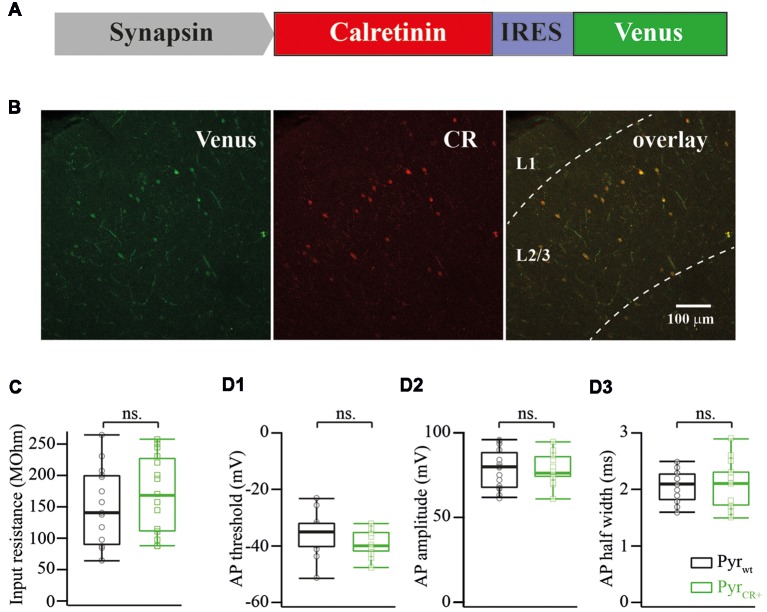
**(A)** Schematic drawing of a viral cassette carrying calretinin and Venus. **(B)** Fluorescent images of a fixed cortical slice infected with AAV carrying calretinin and Venus. Left panel, Venus fluorescence; central panel, immunostaining against calretinin; right panel, overlay of both images. **(C)** Calretinin expression does not change the input resistance of layer 2/3 pyramidal cells (median WT vs. CR+: 143 vs. 173 MOhm; *n* = 15; *p* = 0.319 Mann-Whitney Test rank sum test). **(D)** Calretinin expression does not change the action potential (AP) properties of layer 2/3 pyramidal cells (median WT vs. CR+; *n* = 15 Mann-Whitney rank sum test): **(D1)** AP threshold −35 vs. −40 mV, *p* = 0.12; **(D2)** AP amplitude 79 vs. 76 mV, *p* = 0.9; **(D3)** AP half width 2.1 vs. 2.1, *p* = 0.9, ns, non-significant.

Total mouse mRNA from the forebrain was obtained with TRI Reagent (MRC) extraction and converted to cDNA with the reverse transcription reaction using a mixture of random oligonucleotides (N6) and 20-mer oligo(dT) primers._ORF of the calretinin precursor was amplified from cDNA with a pair of primers: ATGGATCCTTACACGGGGGGCTCACTGC and ATGCTAGCGCCGCCACCATGGCTGGCCCGCAGCAG. The PCR fragment was cloned into a pBluescript vector and sequenced from both ends. Clones confirmed with the proper sequence were used for further cloning; the fragment was excised with NheI and BamHI restriction enzymes and cloned into the pAAV-Syn-IRES-Venus plasmid (Figure 1A).

### Injection

An AAV cassette carrying mouse calretinin-IRES-Venus under a synapsin promoter was packed into AAV using a mix of pDp1 and pDp2 packing vectors (Grimm et al., 2003). The viral suspension was concentrated using a HiTrap Heparin HP affinity column (GE Health Care, Uppsala, Sweden). At P0-P1, rat pups were anesthetized using cold, and two injections (1 μl per site) were made in the somatosensory cortex as previously described (Pilpel et al., 2009). At P14–P23, the injected rat pups were decapitated and the injected half of the neocortex was used to prepare 300-μm-thick parasagittal cortical slices.

### Patch Clamp Recording

Neocortical brain slices were prepared and stored as described (Valiullina et al., 2016). Patch electrodes for both cells were filled with a solution which consisted of (in mM) K-gluconate, 140; KCl, 5; HEPES, 10; NaCl, 8; MgATP, 4; GTP, 0.3; and phosphocreatine, 10 (pH 7.3 with KOH). ACSF contained (in mM) 125 NaCl, 2.5 KCl, 25 glucose, 25 NaHCO_3_, 1.25 NaH_2_PO_4_, 2 CaCl_2_, and 1 MgCl_2_ (carboxygenated with 5% CO_2_/95% O_2_). The infected layer 2/3 pyramidal cells were identified using Venus fluorescence and IR-DIC imaging. In paired recordings, postsynaptic putative FS basket cells in layer 2/3 were identified by location, morphology, and firing pattern (Reyes et al., 1998). All experiments were carried out at room temperature (23–25°C). Signals were recorded using a MultiClamp 700B amplifier, filtered at 3 kHz, and digitized at 10 kHz using a Digidata 1440a (Molecular Devices, USA) and Clampex 10.5 acquisition software (Molecular Devices/Axon Instruments, San Jose, USA).

Synaptic delay was measured as the time difference between an action potential (AP) peak and the onset of the corresponding postsynaptic EPSP, then the values obtained for the individual cell pairs were averaged to get the final estimate.

After electrophysiological recordings, slices were fixed in 4% paraformaldehyde and stained using anti-calretinin antibodies (Swant Inc., dilution 1:1,000). Figure 1B shows an image of an infected neocortex stained with anti-calretinin antibodies. Image analysis of images showed that Venus expression always colocalized with calretinin in layer 2/3 (*n* = 52 cells).

**Figure 2:**
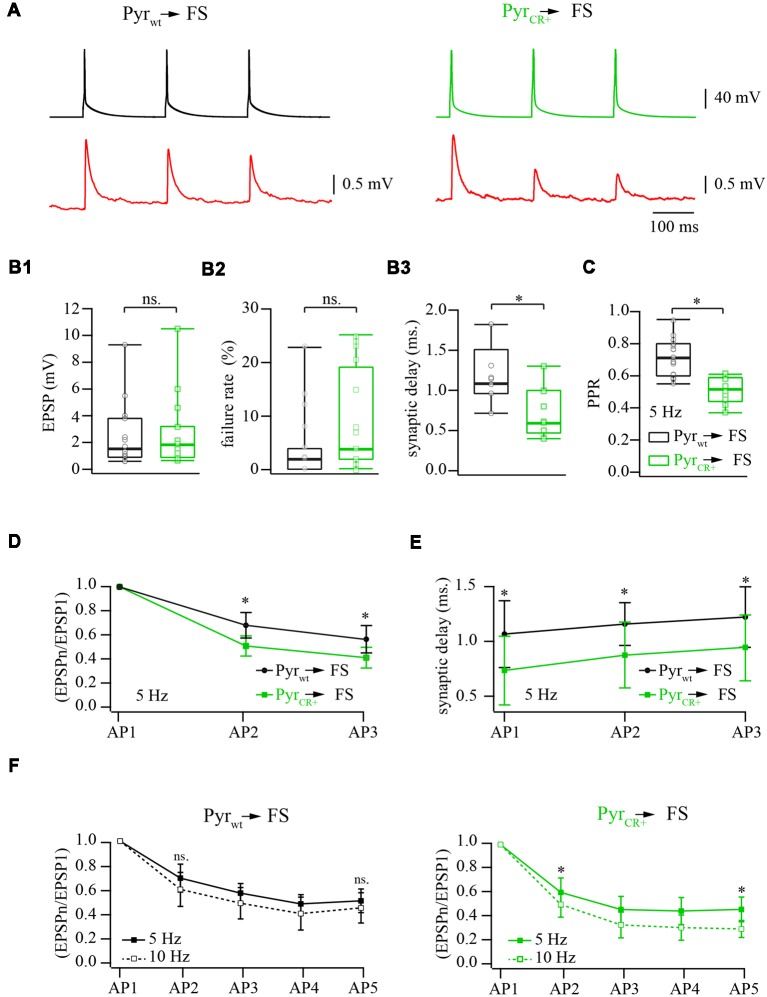
Effect of calretinin expression in pyramidal cells, on the characteristics of layer 2/3 pyramidal neuron to fast-spiking (FS) interneuron synapses. **(A)** Representative averaged traces of presynaptic APs and postsynaptic EPSPs recorded from connected pairs of layer 2/3 pyramidal and FS neurons. Recordings obtained from WT neurons are shown on the left panel, the data from the pairs where presynaptic pyramidal cell was expressing calretinin are on the right. **(B)** Box plots show the effect of the calretinin expression (green box) on the basic release properties (**B_1_**: EPSP amplitudes; **B_2_**: failure rate; **B_3_**: synaptic delay) in comparison to the WT data (black box). Open symbols represent the averaged value obtained in individual experiments. **(C)** Presynaptic calretinin expression reduces paired-pulse ratio (PPR) at layer 2/3 pyramidal to FS cell synapses (green box) relative to WT buffer-free synapses (black box). Open symbols represent the averaged value obtained in individual experiments. **(D)** Comparison of PPR for the second and the third responses in WT (calretinin-free; black) and calretinin-expressing (green) synapses. Data are shown as mean ± SD. **(E)** Reduction of synaptic delay remains significant for the second and the third responses in WT (calretinin-free; black) and calretinin-expressing (green) synapses. Data are shown as mean ± SD. **(F)** Differential effect of simulation frequency enhancement (from 5 to 10 Hz) on “immediate” (EPSP_2_/EPSP_1_) and steady-state depression (EPSP_n_/EPSP_1_) at WT (left panel) and calretinin-expressing synapses (right). Data are shown as mean ± SD. Significant difference is marked as (*), ns, non-significant.

The statistical significance of differences was assessed with the Mann-Whitney rank sum test for two groups with unequal sample sizes and with the paired Student’s *t*-test for pairwise comparison (for data shown on Figure 2F). The level of significance was set at *P* < 0.05. The data are presented as medians and 25th/75th percentile, unless otherwise stated.

## Results

### The Effect of Calretinin Expression on Intrinsic Properties and Excitability of Layer 2/3 Pyramidal Cells and Basic Release Properties at Layer 2/3 Pyramidal to FS Cell Synapses

First, we analyzed the consequences of calretinin overexpression on input resistance, AP threshold, AP amplitude and AP half-width of layer 2/3 pyramidal cells. None of the analyzed features were affected by calretinin expression. The pooled data from 15 calretinin-negative (WT) and 15 virally modified calretinin-positive (CR+) neurons are summarized in Figure 1C,D1–3, with exact median values (WT vs. CR+) provided in the figure legend.

To assess the effect of calretinin expression on basic synaptic properties we measured and analyzed the EPSP amplitude, synaptic delay, and failure rate at synapses between pyramidal cells and FS interneurons in layer 2/3 of the somatosensory cortex. Neurons were identified by their location, cell body shape (observed using infrared microscopy) and their firing pattern. Calretinin-expressing pyramidal cells were recognized by the presence of a fluorescence signal. Postsynaptic responses in the FS cells were triggered by 5 and 10 Hz trains of APs evoked in the presynaptic pyramidal neurons. At least 50 sweeps were collected in each neuronal pair for subsequent analysis (Figure 2A). First, we evaluated synaptic efficacy at the connections formed by WT and CR+ pyramidal neurons onto FS cells. To this end, we compared the averaged amplitudes of the first EPSP in the trains. The median values were very similar being 1.64 mV (*n* = 12) and 1.98 mV (*n* = 12) in WT and CR+ cell pairs respectively, (*p* = 0.42; Mann-Whitney rank sum test; Figure 2B1). This result was expected, since prior to every AP train calretinin was not bound to Ca^2+^ and, therefore, was acting as a slow buffer. In agreement with this, the failure rate was slightly increased in the CR+ pyramidal cells, but this enhancement was not significant. The median value of failure rate in WT pyramidal cell terminals was 2% (*n* = 12) and, in CR+ terminals, 7.5% (*n* = 10, *p* = 0.3; Mann-Whitney rank sum test; Figure 2B2). However, even a slow buffer can shorten the temporal and spatial spread of the calcium microdomains and consequently reduce synaptic delay. Indeed, EPSPs recorded from the pairs where presynaptic pyramidal cell express calretinin were significantly better synchronized with APs as indicated by the shorter synaptic delays. Median values were 1.08 (*n* = 7) and 0.6 (*n* = 9) milliseconds at WT and CR+ synapses, respectively (*p* = 0.026; Mann-Whitney rank sum test; Figure 2B3).

### The Effect of Calretinin Expression on Short-Term Plasticity at Layer 2/3 Pyramidal to FS Cell Synapses

The binding of the first Ca^2+^ ion to calretinin strongly increases the affinity of the remaining unoccupied binding sites converting calretinin from a slow calcium buffer to a fast calcium buffer. It has been shown that at layer 2/3 pyramidal to FS cell synapses slow buffers (EGTA) do not have a strong impact on short-term depression; this is in contrast with the fast buffer BAPTA which strongly reduces the amplitude of the first response and degree of depression (Rozov et al., 2001). Therefore, next we tested how the transition from the slow to fast Ca^2+^ binding modes of calretinin affects short-term plasticity at these synapses. In WT animals the median value of paired-pulse ratio (PPR) of the second and the first EPSPs evoked by 5 Hz trains of three APs was 0.71 (*n* = 17), however, PPR measured at CR+ synapses was significantly smaller, 0.52 (*n* = 10; *p* = 0.0009; Mann-Whitney rank sum test; Figure 2C). Significant enhancement of depression in the presence of calretinin persisted for the third EPSP (*p* = 0.005; Mann-Whitney rank sum test; Figure 2D). Similarly to the first EPSP, the second and the third responses were more synchronized with APs in pairs formed by calretinin expressing pyramidal cells (EPSP2, *p* = 0.008; EPSP3, *p* = 0.009; Mann-Whitney rank sum test; Figure 2E).

At WT layer 2/3 pyramidal to FS cell synapses, a change in stimulation frequency from 5 to 10 Hz results in moderate and often not significant enhancement of both paired-pulse and steady state depression (Beck et al., 2005). However, at higher stimulation frequencies the ratio between the “slow” and “fast” forms of calretinin should be shifted towards the latter due to an increased level of residual intraterminal Ca^2+^ concentration. Taking into account that “fast” calretinin promotes paired-pulse depression, we compared the frequency-dependent dynamics of EPSPs evoked by 5 and 10 Hz trains of five APs elicited in the same cell pairs. In WT animals, depression was slightly more pronounced for all APs in the train but these changes were not significant (*n* = 6; *p* > 0.05 paired Student’s *t*-test; Figure 2F). Contrarily, at CR+ synapses, the increase in the stimulation frequency caused strong enhancement of both paired-pulse and steady state depression. The EPSP2/EPSP1 ratios recorded at 5 and 10 Hz were 0.6 ± 0.11 and 0.5 ± 0.1, respectively, (*n* = 7; *p* = 0.04 paired Student’s *t*-test; Figure 2F). At steady-state level, during the fifth AP, the difference between ratios were even more pronounced (EPSP5/EPSP1 at 5 Hz 0.46 ± 0.1 and at 10 Hz 0.29 ± 0.07; *p* = 0.013 paired Student’s *t*-test). Thus, in contrast to another fast-endogenous buffer, calbindin which has the main influence on the synaptic release during the first AP in the train and after partial saturation with Ca^2+^ can cause synaptic facilitation, the calretinin effect on the first response is rather weak. However, “sensitization” of calretinin by Ca^2+^ during the first AP increases its binding properties and therefore leads to the suppression of synaptic release.

## Discussion

Synaptic transmission requires presynaptic Ca^2+^ entry *via* voltage-gated Ca^2+^ channels (VGCC) and subsequent vesicle fusion is triggered by Ca^2+^ binding to the release calcium sensor. Synaptic release occurs at active zones in the close vicinity of presynaptic VGCC where [Ca^2+^]_i_ rapidly rises reaching levels sufficient for activation of exocytosis in a very short time. This spatially and temporally restricted elevation of [Ca^2+^]_i_ is known as a Ca^2+^-concentration microdomain. Conversion of Ca^2+^ entry into characteristic release properties (release probability, synaptic delay and short-term plasticity mode) at individual types of synapses crucially depends on diffusional distance, as well as on endogenous buffer capacity and buffering kinetics. It is commonly accepted that release probability (*Pr*) is negatively related to the distance between VGCCs and Ca^2+^ vesicular release sensors (Neher, 1998), while synaptic latency is usually directly proportional to the diffusional distance (Rozov et al., 2001). In synapses, sparsely distributed VGCC and low endogenous buffer capacity accumulation of free residual Ca^2+^ during frequency stimulation often leads to facilitation. In terminals where VGCC are more tightly coupled to the Ca^2+^ sensor, high levels of [Ca^2+^]_i_ at the release site result in greater synchronization of release with APs and higher *Pr*. Synapses with a short diffusional distance are usually characterized by pronounced short-term depression (Neher, 1998). The effect of Ca^2+^ buffers on synaptic release in these two types of terminals depends on their binding kinetics. Slow Ca^2+^ buffers like exogenous EGTA and endogenous parvalbumin can efficiently reduce synaptic efficacy and block facilitation at low*-Pr* synapses and do not have a significant effect on the amplitude of the postsynaptic responses and short-term plasticity at synapses with high-*Pr*. Buffers with fast binding kinetics (BAPTA or calbindin) can bind Ca^2+^ before it reaches vesicular sensors, and therefore, significantly reduce *Pr* at both types of terminals. The effect of a given buffer concentration is several fold stronger at low-*Pr* compared to high-*Pr* synapses. However, during repetitive high frequency presynaptic activity, fast buffers undergo partial saturation with Ca^2+^ which attenuates the suppressant effect on release and leads to synaptic facilitation or a significant reduction in paired pulse depression (Rozov et al., 2001; Blatow et al., 2003; Voinova et al., 2015).

In contrast to calbindin, at low [Ca^2+^]_i_ calretinin behaves like a typical slow buffer with a binding rate lower than in EGTA (CR: *k*_on_ 1.8 μM^−1^s^−1^; EGTA: *k*_on_ 10 μM^−1^s^−1^). However, when the first pair of binding sites is occupied with Ca^2+^, *k*_on_ of the second pair drastically increases to 310 μM^−1^s^−1^ (Faas et al., 2007). For comparison, the fast buffer BAPTA has *k*_on_ 400 μM^−1^s^−1^ (Naraghi and Neher, 1997; Meinrenken et al., 2002). Faas et al. (2007) described this phenomenon in terms of the positive cooperativity as ability to increase the ligand binding affinity of one site of a macromolecule by previous binding at another site of the same ligand to the same molecule. Hence, during the first AP in the train when [Ca^2+^]_i_ is in the range of 100 nM, calretinin acts as a slow buffer and does not have a significant effect on release at least in high-*Pr* terminals. Indeed, both failure rate and amplitude of the unitary EPSPs were very similar in WT and calretinin expressing pyramidal cells. In addition, it suggests that in CR+ synapses the concentration of calretinin does not exceed a few hundred micromoles. However, AP-driven Ca^2+^ entry (in the range of 5–20 μM in close vicinity to the VGCC) can partially saturate the first binding site of calretinin shifting its Ca^2+^ binding affinity from “slow calretinin” to the “fast 1Ca^2+^-calretinin”. “Fast” calretinin in turn can more effectively prevent Ca^2+^ binding to the Ca^2+^ sensor release suppressing vesicle fusion more efficiently during the second AP. Upon long lasting repetitive stimulation, the rate of “partial saturation” and, therefore, the ratio between “slow” and “fast” calretinins will depend on K_off_ of 1Ca^2+^-calretinin and 2Ca^2+^-calretinin, calretinin diffusion rates, and most probably, on residual [Ca^2+^]_i_ and the Ca^2+^ extrusion rate. There is evidence suggesting that residual [Ca^2+^]_i_ (>1 μM) remains in the terminals for hundreds of milliseconds after AP (Koester and Sakmann, 2000). Consequently, during bursts of presynaptic activity that are not long enough to cause complete saturation of calretinin, frequency-dependent accumulation of residual [Ca^2+^]_i_ can accelerate a transition from the “slow” calretinin to the “fast” 1Ca^2+^-calretinin resulting in stronger depression. However, in this case, the total Ca^2+^-binding capacity of the buffer in the terminal should considerably exceed the amount of Ca^2+^ coming with each AP. Thus, upon high frequency stimulation calretinin has a moderate effect on synaptic transmission during the first AP, but can greatly reduce information flow throughout the rest of the train.

## Data Availability

All datasets generated for this study are included in the manuscript.

## Author Contributions

All authors listed have made a substantial, direct and intellectual contribution to the work, and approved it for publication.

## Conflict of Interest Statement

The authors declare that the research was conducted in the absence of any commercial or financial relationships that could be construed as a potential conflict of interest.
